# 864 10-Year National Database Review of Pneumothorax After Burn Injuries

**DOI:** 10.1093/jbcr/iraf019.395

**Published:** 2025-04-01

**Authors:** Hilary Liu, Daniel Najafali, Christopher Fedor, José Arellano, Rebecca Hohsfield, Mare Kaulakis, Garth Elias, Alain Corcos, Jenny Ziembicki, Francesco Egro

**Affiliations:** University of Pittsburgh Medical Center; Carle Illinois College of Medicine; University of Pittsburgh School of Medicine; University of Pittsburgh Medical Center; University of Pittsburgh Medical Center; University of Pittsburgh School of Medicine; University of Pittsburgh Medical Center; University of Pittsburgh Medical Center; University of Pittsburgh Medical Center; University of Pittsburgh Medical Center

## Abstract

**Introduction:**

Pneumothorax is a recognized complication in burn patients, particularly those with inhalation injuries, chest wall burns, or requiring mechanical ventilation. Despite its clinical significance, there is limited descriptive data on its incidence and characteristics in this population. This study aims to describe pneumothorax in burn patients, with a focus on its frequency, associated factors, and outcomes.

**Methods:**

A retrospective review was performed using data from the ABA National Registry from January 2013 to December 2022 on burn patients who developed pneumothorax. Patient demographics, burn characteristics, clinical course, and hospitalization data were extracted and analyzed using descriptive statistics.

**Results:**

A total of 258 burn patients developed pneumothorax. The mean age was 47.9 ± 23.1 years. The majority of patients were male (n=163; 63.2%) and white (n=166; 64.3%). 11.6% (n=30) had diabetes mellitus, 3.5% (n=9) had chronic obstructive pulmonary disease (COPD), and 24.0% (n=62) were current smokers. The mean total body surface area (TBSA) burned was 31.5 ± 24.0%. Flame burns were the most common etiology (n=169; 65.5%), followed by scald (n=17; 6.6%) and flash burns (n=14; 5.4%). Inhalation injury was present in 40.3% (n=104) of cases. The most frequent place of occurrence was private residences (n=169; 65.5%).

The mean initial Glasgow Coma Scale (GCS) score was 9.7 ± 5.4, and the mean initial emergency department temperature was 36.3 ± 1.4°C. The mean carboxyhemoglobin level, when measured, was 7.0 ± 9.2%. The mean hospital length of stay was 54.8 ± 55.6 days. Patients spent an average of 43.4 ± 48.9 days in the ICU and 29.3 ± 38.1 days on mechanical ventilation. Acute renal failure requiring renal replacement therapy occurred in 13.9% (n=36) of patients. The overall mortality rate was 25.6% (n=66). Among survivors, the most common discharge dispositions were transfer to another hospital (n=96; 37.2%) and discharge to home or self-care (n=58; 22.5%).

**Conclusions:**

Pneumothorax mainly affects middle-aged males with flame burns and inhalation injuries, resulting in longer ICU stays, increased ventilation duration, and high mortality rates.

**Applicability of Research to Practice:**

The results can inform risk stratification, treatment protocols, and outcome prediction for pneumothorax in burn patients. Future research should focus on prevention and management in high-risk groups, particularly those with inhalation injuries, and address the need for comprehensive care and home safety strategies.

**Funding for the Study:**

N/A

